# MRI adenosine fist-pass perfusion analysis using a SSFP sequence – are there gender differences?

**DOI:** 10.1186/1532-429X-11-S1-P232

**Published:** 2009-01-28

**Authors:** Nico Merkle, Markus Kunze, Volker Rasche, Matthias Kochs, Vinzenz Hombach, Jochen Woehrle

**Affiliations:** University Ulm, Ulm, Germany

**Keywords:** Significant Gender Difference, Coronary Artery Stenosis, Luminal Narrowing, Myocardial Scar, Consecutive Cohort

## Purpose

Significant gender differences have been found in the performance of exercise ECG for the identification of coronary artery disease. Our aim was to evaluate possible differences in the diagnostic power of cardiac MRI using SSFP-Perfusion under adenosine related vasodilatation in both gender subgroups in a large consecutive cohort of patients with suspected CAD.

## Methods

228 (male n = 180, female n = 48) patients with suspected CAD were examined with MRI (1.5 T Intera CV) and coronary angiography. A 3-slice short axis SSFP perfusion scan with a saturation prepulse before each slice was performed during infusion of adenosine and at rest followed by myocardial scar imaging. Gadolinium – DTPA was given at 0.1 mmol/kg body weight. Images were assessed visually by two observers in a joint reading.

## Results

Sensitivity, specificity and accuracy of MRI first-pass perfusion for detection of a coronary artery stenosis (>50% luminal narrowing) on a per patient basis were 92,3%, 81,6% and 90.0% in the male subgroup and 97,7%, 94,4% and 95,8% in the female subgroup. The results for detection of a significant lesion (>70% luminal narrowing) were 96.0%, 68,5% and 87,8 in the male subgroup and 96,3%, 80,9% and 89,6% in the female subgroup.

## Conclusion

In contrast to data from stress ECG, MRI adenosine fist-pass perfusion analysis using a SSFP sequence reveals no differences regarding diagnostic performance in female compared to the male group in our cohort.

